# Estimating the cost-per-result of a national reflexed Cryptococcal antigenaemia screening program: Forecasting the impact of potential HIV guideline changes and treatment goals

**DOI:** 10.1371/journal.pone.0182154

**Published:** 2017-08-22

**Authors:** Naseem Cassim, Lindi Marie Coetzee, Kathryn Schnippel, Deborah Kim Glencross

**Affiliations:** 1 National Health Laboratory Service (NHLS), National Priority Programmes, Johannesburg, South Africa; 2 Department of Molecular Medicine and Haematology, Faculty of Health Sciences, University of Witwatersrand, Johannesburg, South Africa; 3 Right to Care, Johannesburg, South Africa; 4 Department of Clinical Medicine, Faculty of Health Sciences, University of Witwatersrand, Johannesburg, South Africa; Saint Louis University, UNITED STATES

## Abstract

**Introduction:**

During 2016, the National Health Laboratory Service (NHLS) introduced laboratory-based reflexed Cryptococcal antigen (CrAg) screening to detect early Cryptococcal disease in immunosuppressed HIV+ patients with a confirmed CD4 count of 100 cells/μl or less.

**Objective:**

The aim of this study was to assess cost-per-result of a national screening program across different tiers of laboratory service, with variable daily CrAg test volumes. The impact of potential ART treatment guideline and treatment target changes on CrAg volumes, platform choice and laboratory workflow are considered.

**Methods:**

CD4 data (with counts < = 100 cells/μl) from the fiscal year 2015/16 were extracted from the NHLS Corporate Date Warehouse and used to project anticipated daily CrAg testing volumes with appropriately-matched CrAg testing platforms allocated at each of 52 NHLS CD4 laboratories. A cost-per-result was calculated for four scenarios, including the existing service status quo (Scenario-I), and three other settings (as Scenarios II-IV) which were based on information from recent antiretroviral (ART) guidelines, District Health Information System (DHIS) data and UNAIDS 90/90/90 HIV/AIDS treatment targets. Scenario-II forecast CD4 testing offered only to new ART initiates recorded at DHIS. Scenario-III projected all patients notified as HIV+, but not yet on ART (recorded at DHIS) and Scenario-IV forecast CrAg screening in 90% of estimated HIV+ patients across South Africa (also DHIS). Stata was used to assess daily CrAg volumes at the 5^th^, 10^th^, 25^th^, 50^th^, 75^th^, 90^th^ and 95^th^ percentiles across 52 CD4-laboratories. Daily volumes were used to determine technical effort/ operator staff costs (% full time equivalent) and cost-per-result for all scenarios.

**Results:**

Daily volumes ranged between 3 and 64 samples for Scenario-I at the 5th and 95th percentile. Similarly, daily volumes ranges of 1–12, 2–45 and 5–100 CrAg-directed samples were noted for Scenario’s II, III and IV respectively. A cut-off of 30 CrAg tests per day defined use of either LFA or EIA platform. LFA cost-per-result ranged from $8.24 to $5.44 and EIA cost-per-result between $5.58 and $4.88 across the range of test volumes. The technical effort across scenarios ranged from 3.2–27.6% depending on test volumes and platform used.

**Conclusion:**

The study reported the impact of programmatic testing requirements on varying CrAg test volumes that subsequently influenced choice of testing platform, laboratory workflow and cost-per-result. A novel percentiles approach is described that enables an overview of the cost-per-result across a national program. This approach facilitates cross-subsidisation of more expensive lower volume sites with cost-efficient, more centralized higher volume laboratories, mitigating against the risk of costing tests at a single site.

## Introduction

HIV-infected individuals, with a CD4 count below 100cells/μl, are most susceptible to opportunistic infections, i.e. tuberculosis and cryptococcal disease (caused by *Cryptococcus neoformans)*. Screening for Cryptococcal antigenaemia (CrAg), followed by pre-emptive treatment if detected, can reduce Cryptococcal disease (CD) related morbidity and mortality [1–3]. Detection of CD in predisposed HIV+ immunosuppressed patients, can be implemented in one of two ways: (i) provider-initiated screening where the attending clinician orders a CrAg test, based on either clinical presentation and/or confirmed CD4 count <100cells/μl; this screening is typically done in a Microbiology laboratory [4]. Alternatively, (ii) a reflexed laboratory-based screening approach can be implemented where routine CD4 testing is offered. Remnant CD4 samples are automatically screened for CrAg if a CD4 count below 100cells/μl is confirmed. Reflexed screening enables the CrAg result to be reported simultaneously with the originating CD4 count, to affect prompt clinical intervention if CrAg is detected.

In 2012, the National Institute of Communicable Diseases (NICD), in collaboration with the National Health Laboratory Service (NHLS), launched a pilot reflexed CrAg screening project in three CD4 laboratories. Samples from local designated HIV/AIDS health facilities were screened for CrAg using a manual lateral flow assay (LFA) [5] (IMMY Mycologics, USA). By June 2016, the pilot extended CrAg screening to include a further six CD4 laboratories serving ~ 500 pre-selected health facilities across four provinces. During the pilot study, a cost-per-result was established [6] to facilitate local budgetary planning and enable a collaborative cost-effectiveness analysis [7] which revealed that reflexed CrAg screening was cost-effective and saved more lives compared to provider-initiated testing. An integrated tiered service delivery model (ITSDM) was implemented in the NHLS for CD4 testing [8] that provides for five tiers of service based on daily volumes that dictate laboratory tier and platform allocation [8]. It is anticipated that a national reflexed CrAg screening service would follow suit utilising commercially available systems that match workloads at different test volumes.

It is understood that laboratory test costs can vary according to choice of testing platform, reagent costs, workload, staff allocation, capital cost overheads and local currency exchange fluctuation, all of which need to be factored in when establishing a cost-per-result. The work presented here builds on earlier costing done in a single busy CD4 laboratory using of the LFA assay [6]. The aim of this study is to provide further insights into anticipated costs expected across a national CrAg screening program in light of forecasted changes in test volumes due to local and international guideline changes and scale up initiatives like the 90-90-90 targets [9–12]. These are expected to impact the volume of tests required which will dictate testing platform choice. Additional test scenarios were defined for the purpose of this study to predict the outcome on a cost-per-result in an HIV/ AIDS environment where test volumes could change significantly.

## Methods

### Establishing workload

The 2015/16 CD4 volumes with counts < = 100 cells/μl were used to calculate monthly and daily CrAg screening volumes for each of 52 CD4 laboratories, assuming 12 months per year and 22 working days per calendar month. The percentage (as a proportion of total) of CD4 <100 cells/μl, was calculated for each laboratory (n = 52). The individual laboratory’s percentage of the national workload was then allocated to predict anticipated CrAg testing volumes per laboratory for the scenarios described below. For example, in a selected scenario, laboratory A contributed 7.2% of 2015/16 national CD4 < = 100 cells/μl workload, whereas another laboratory, X, where the workload was considerably smaller, contributed just 0.1% of national test volumes.

Predicted daily CrAg volumes were derived and rounded up using Microsoft Excel. Stata was used to calculate defined percentiles for daily CrAg volumes at the 5th, 10th, 25th, 50th, 75th, 90^th^ and 95th percentiles across 52 laboratories for the four scenarios described below.

### Defining scenarios for screening services evaluation of costs

Four scenarios were investigated. The first, ‘Scenario I’ is based on the existing South African laboratory CD4 service status quo which provided ~3.4 million CD4 tests during 2015/16, in line with local and international HIV/ AIDS guidelines for treating patients enrolled for care [10, 13]. The current CD4 volumes consist of both patients being screened in the pre-ART wellness program as well as monitoring those already on treatment.

Three additional scenarios were developed to emulate possible guideline changes that could markedly affect CrAg volumes across a national screening program. This included both scaling up and scaling down of CrAg services. Scenarios II–IV, were based on information distributed in the 7th South Africa AIDS Conference HIV/AIDS factsheet [9] reported from District Health Information System (DHIS) data and the Joint United Nations Program on HIV/AIDS United Nation (UNAIDS) 90/90/90 HIV/AIDS antiretroviral treatment initiative targets [11].

**Fig 1:**
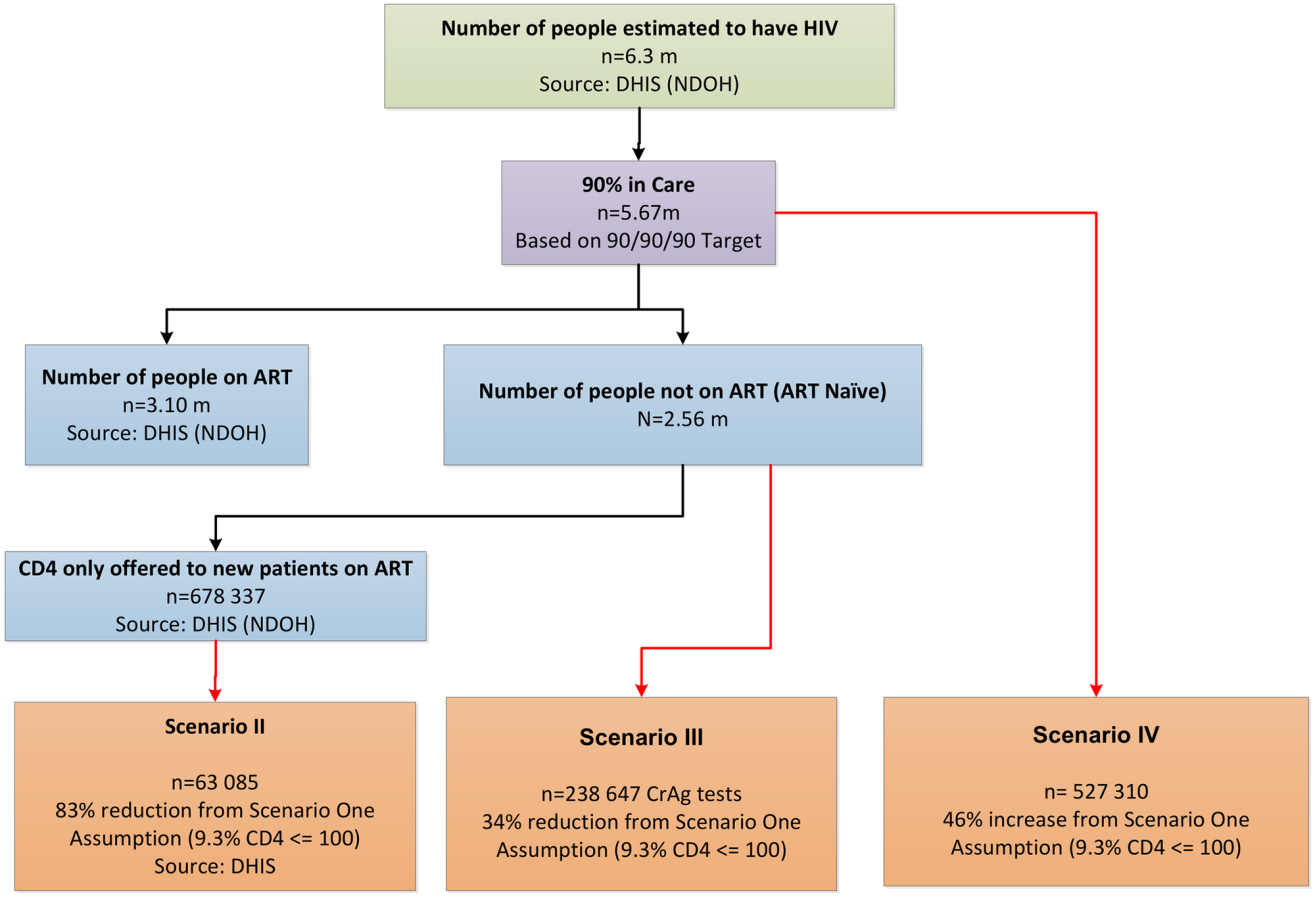
Decision tree. A descriptive decision tree used to forecast annual CrAg volumes for Scenarios II, III and IV based on the National Department of Health (NDOH) HIV guidelines, UNAIDS 90/90/90 HIV/ AIDS treatment targets and published DHIS data.

The description of the three additional scenarios follows: (1) ‘Scenario-II’, CD4 is a setting where CD4’s are performed only for new patients initiated on ART, i.e. patients who have enrolled for HIV counseling and treatment (HCT) and who have recently started ART (early ART initiates); (2), ‘Scenario-III’ is a setting where all known HIV-positive individuals across South Africa, who have not yet been initiated on ART, are screened. Lastly, (3), ‘Scenario-IV’, provides for wide-scale 90-90-90 scale-up of local HCT services to include CD4 testing and associated CrAg screening for at least 90% of all estimated HIV positive patients in South Africa. Further explanation of the respective decision tree and how the scenarios were constituted, can be seen in Fig 1.

### Managing reflexed CrAg screening workload: Allocating testing platforms

Across all scenarios described, a reflexed CrAg screening laboratory service is provided using two appropriately selected, commercially-available platforms to facilitate and match daily workloads. In busy CD4 laboratories, an automated walk-away enzyme immunoassay (EIA) platform [14] with primary tube sampling and laboratory information system (LIS) interfacing, is allocated (using the CrAg EIA kit, IMMY Mycologics, USA and the Thunderbolt automated EIA analyser, Gold Standard Diagnostics, USA). In lower volume laboratories, the manual LFA test (IMMY Mycologics, USA) [5] was allocated as CrAg assay of choice. For the purpose of this study, based on earlier workload analysis, a cut-off of 30 samples per day was used to allocate either EIA (>30) versus the LFA (< = 30) testing platforms. This cut-off ensures that CrAg testing can be included in the CD4 routine laboratory without the need for additional staff to manage workload.

### Costing methodology

The costing analysis was undertaken using Microsoft Excel and Stata software. Annual predicted laboratory volumes according to defined percentiles (5^th^, 10^th^, 25^th^, 50^th^, 75^th^, 90^th^ and 95^th^) were used to derive annual equivalent cost-per-result. Discrete costs considered included laboratory equipment, reagents and technical effort (cost of staffing personnel required to perform the testing in the laboratory). These were obtained using expenditure data from the NHLS Oracle Enterprise Resource Planning (ERP) system, as well as manufacturer-supplied quotations. A provider prospective was assumed with all costs reported with the NHLS as the CrAg screening service provider. All costs are reported in USD using an exchange rate of R14.43 as at 11 July 2016 [15]. Organizational overheads, buildings, logistics and infrastructure costs as well as pre-analytical processing costs were excluded as these costs are already included in the CD4 test in a reflexed testing context. Costs related to activities such as training, monitoring and support site visits and provision for External Quality Assessment (EQA) program participation were also not assessed. The Consolidated Health Economic Evaluation Reporting Standards (CHEERS) checklist was used in the preparation of the manuscript (19).

#### Workflow analysis and technical effort/ staffing costs

An actual stopwatch timing exercise was done to assess the individual steps to perform CrAg testing using either LFA or EIA platforms (S1 Table). This exercise was repeated for varying daily volumes at defined percentiles across the four scenarios described above. For the LFA platform, it was assumed that staff would be unable to multitask due to the nature of performing a manual hands-on LFA assay with short manufacturer incubation times. Effort recorded (S1 Table) included time spent on preparing the laboratory information system (LIS) work list and locating CD4 samples earmarked for reflexed CrAg screening, preparing test tubes, adding diluents and controls, adding patient plasma (40μl), inserting the LFA test strip, observing a 10min incubation period, reading and recording results on the work list, capturing and reviewing results on the LIS and re-filing screened samples (S1 Table). During automated EIA testing, it was assumed that staff would continue with general CD4-based laboratory activities during automated EIA analysis. Here, minimal effort was recorded for sample preparation, analysis and resulting as these are automated steps, but time is still needed on the administrative aspects of locating CD4 samples earmarked for CrAg screening, i.e. time is required to generate the work list, locate CD4 samples and reviewing and re-filing samples (S1 Table).

NHLS mid-point cost-to-company (CTC) salary scales for a medical technologist (grade C2, entry level) were used to predict technical effort/ staff costs ($28 905 per annum). A percentage of a full time equivalent (FTE) of an attending staff member performing CrAg testing was calculated by taking the total time required to perform a batch of samples (in minutes) at each scenario pre-defined percentile test volumes and dividing this by the number of expected working minutes per day (equivalent to 405 minutes in an 8-hour working day, minus a 75-minute allocation for lunch and morning tea break). Across the four scenarios and defined percentiles, the %FTE required to facilitate the respective workload is reported. Annual technical effort/ staff costs were then established by multiplying the annual CTC salary by %FTE.

#### Reagent costs

Reagents costs were obtained by manufacturer quotation for both the LFA or EIA CrAg kits (50 and 192 tests respectively). Additional test consumables included pipette-tips, gauze, micro-tubes, and gloves for LFA. EIA test consumables included gloves, distilled water, printer cartridge and paper (but not pipettes-tips and gauze as these are not required for EIA).

#### Laboratory equipment costs

A working life of 5 years and a discount rate of 4% were applied (a discount rate between 3 and 5% is recommended for healthcare costing studies [16]) and based on the current term CD4 tender service level agreement. LFA assay equipment costs assessed included only pipettes and specimen tube racks whereas the Thunderbolt EIA analyzer with mandatory service/maintenance contract costs were included under laboratory equipment for the EIA platform.

## Results

### Annual CrAg volumes and allocation of platforms

A marked variation in annual projected CrAg volumes was noted across the four scenarios described (Table 1). In Scenario I, the prevailing service context, annual CrAg test volumes of 344 506 were reported. This number was however projected to reduce by 82% to 63 085 p.a. for Scenario II and by 31%, to 238 647 p.a. in Scenario III. In Scenario IV, in the context of massive scale-up of services to reach 90-90-90 goals (Table 1), total CrAg screening volumes are projected to increase to 537 310 (increasing of 56%) p.a.

**Table 1:** Distribution of the LFA and EIA annual test volumes and testing sites across four scenarios (see Methods for details).

Platform	Scenario One		Scenario Two		Scenario Three		Scenario Four	
**Annual Volumes**	**344 506**	**100%**	**63 085**	**100%**	**238 647**	**100%**	**537 310**	**100%**
EIA	236 859	68.75%	-	0%	81 028	33.95%	429 433	79.92%
LFA	107 647	31.25%	63 085	100%	157 619	66.05%	107 877	20.08%
**Laboratories**	**52**	**100%**	**52**	**100%**	**52**	**100%**	**52**	**100%**
EIA	19	36.54%	-	0%	7	13.46%	25	
LFA	33	63.46%	52	100%	45	86.54%	27	

Allocation of the appropriate CrAg testing platforms based on daily testing volumes per laboratory changed dramatically between scenarios (see Table 1). In Scenario I, 31.25% of annual CrAg test volumes were allocated for testing by LFA platform (33/52 laboratories), with 25 sites allocated the EIA high volume platform (but carrying 68.75% of the national workload). This percentage (as a proportion) increased to 100% (52/52 laboratories) in Scenario II, where all laboratories would have daily CrAg volumes best suited for LFA testing (<30 samples per day) due to substantially reduced CD4 testing volumes for this scenario. Similarly, in Scenario III, the LFA platform was allocated to perform 86.5% of national CrAg test volumes in 45/52 CD4 laboratories, with far fewer sites (7/52) needing automation to cope with daily throughput (just 13.5% of national volumes would be allocated the EIA platform). In Scenario IV, most sites would need to scale up to meet the increased daily volumes projected, with the automated EIA platform required in 25 laboratories, screening 80% of national CrAg tests, while the LFA platform would only perform 20% of annual CrAg screening volumes in27 testing sites.

### Daily CrAg volumes

**Fig 2:**
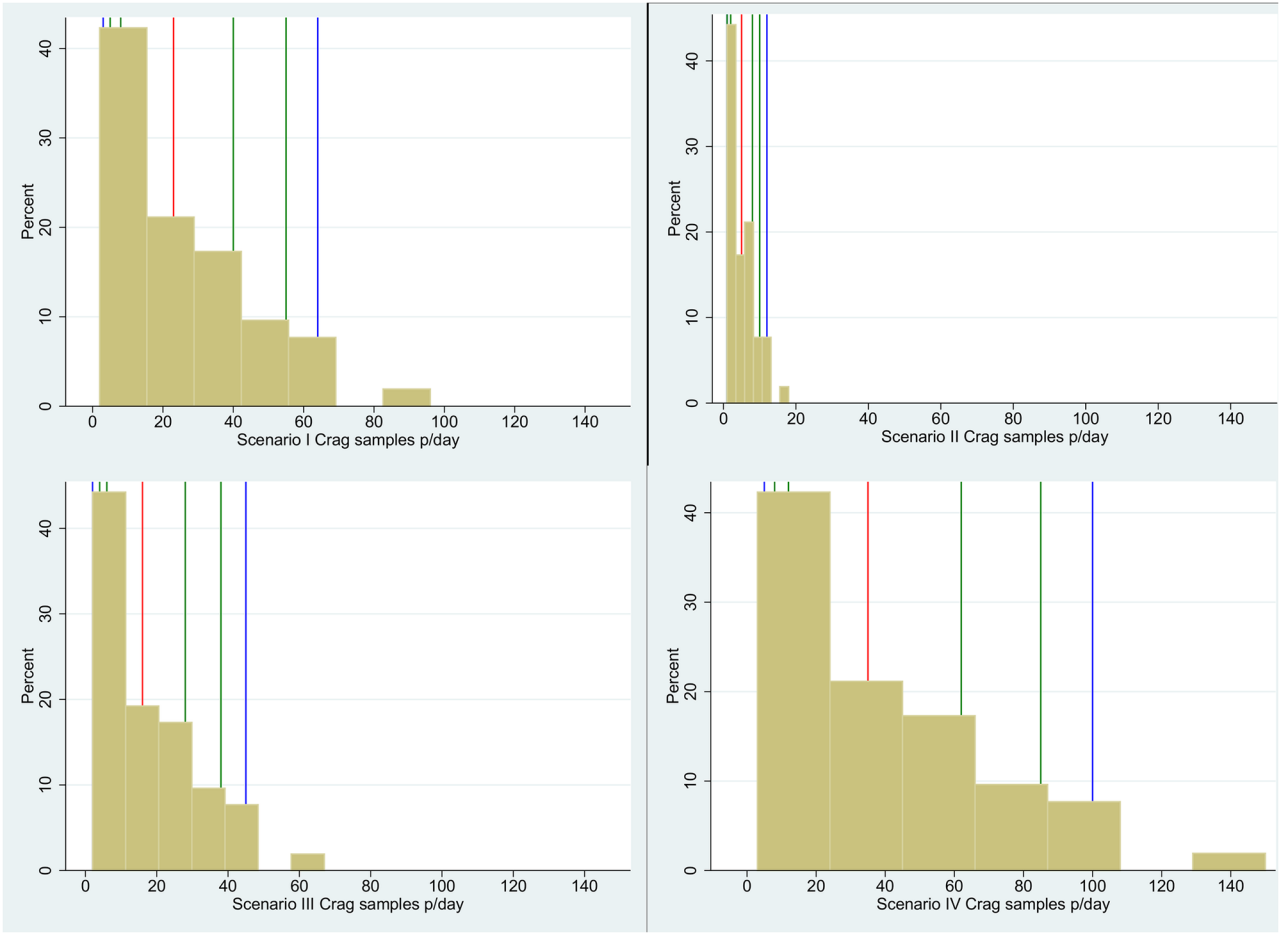
Distribution of test volumes. This figure represents the four test volume scenarios (I-IV) where the x-axis represents the test volumes (fixed across scenarios) and the Y-axis the predicted percentage of laboratories performing these volumes. The red colored lines represent the 50^th^ percentile, while the blue lines represent the 5^th^ and 95^th^ percentiles. The remaining consecutive percentiles are colored green (10^th^, 25^th^, 75^th^, 90^th^ percentiles).

Fig 2 shows the distribution of daily CrAg volumes across 52 laboratories for scenarios I to IV (see Fig 2 and Table 2), noting the 5^th^, 10^th^, 25^th^, 50^th^, 75^th^, 90^th^ and 95^th^ percentiles. In Scenario I, percentile daily volumes reported ranged from 3 to 64 (from 5^th^ through 95^th^ percentiles). For Scenario II, daily CrAg volumes decreased significantly with a range of 1 to 12 samples per day (5^th^ to 95^th^ percentiles), with a small increase in Scenario III, having a range of 2–45 samples per day. For Scenario IV the volumes increased significantly with a range of 5 to 100 samples per day at the reported percentiles.

**Table 2:** Daily CrAg volumes, cost-per-result and %FTE (technical effort) at defined percentiles across four scenarios (see Methods for details).

Percentile	Scenario One				Scenario Two				Scenario Three				Scenario Four			
	Daily CrAg volume	Platform	Cost per Result (USD)	% FTE	Daily CrAg volume	Platform	Cost per Result (USD)	% FTE	Daily CrAg volume	Platform	Cost per Result (USD)	% FTE	Daily CrAg volume	Platform	Cost per Result (USD)	% FTE
**5th Percentile**	3	**LFA**	$6.41	4.6%	1	**LFA**	$8.24	3.2%	2	**LFA**	$6.90	3.9%	5	**LFA**	$5.88	5.7%
**10th Percentile**	5		$5.88	5.7%	1		$8.24	3.2%	4		$6.48	5.3%	8		$5.84	8.2%
**25th Percentile**	8		$5.84	8.2%	2		$6.90	3.9%	6		$5.82	6.7%	12		$5.63	11.0%
**50th Percentile**	23		$5.44	18.5%	5		$5.88	5.7%	16		$5.49	13.5%	35	**EIA**	$5.58	15.4%
**75th Percentile**	40	**EIA**	$5.43	16.3%	8		$5.84	8.2%	28		$5.45	22.4%	62		$5.12	20.0%
**90th Percentile**	55		$5.19	18.8%	10		$5.74	9.6%	38	**EIA**	$5.47	16.0%	85		$4.94	24.4%
**95th Percentile**	64		$5.08	20.4%	12		$5.63	11.0%	45		$5.31	17.1%	100		$4.88	27.5%

### Workflow analysis

**Fig 3:**
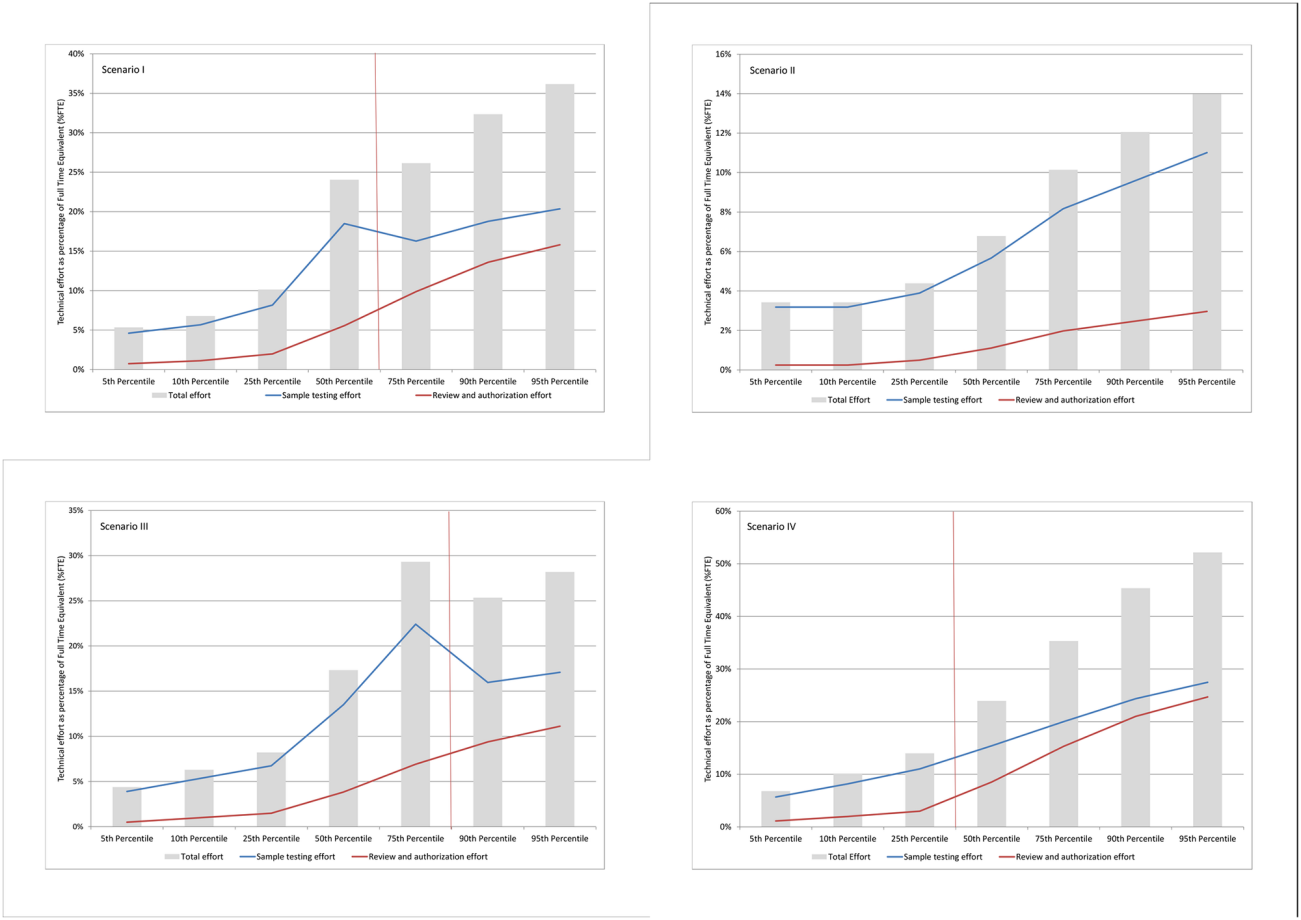
Technical effort required. This figure shows the technical effort required, as a percentage of a Full Time Equivalent (%FTE) for the defined consecutive percentiles (5^th^, 10^th^, 25^th^, 50^th^, 75^th^, 90^th^ and 95^th^). Total technical effort (grey bars), as well as the proportion of the technical effort that is required for actual CrAg testing (blue line) with full review and authorization of results effort indicated as a solid red line.

The technical effort required to produce a reflexed CrAg result was reported as a percentage of a Full Time Equivalent (%FTE) across scenarios I to IV, for both LFA and EIA-based testing sites (see Table 2 and Fig 3; stop-watch timing detail is contained in S1 Table). In Scenario I, laboratory staff would be required to allocate technical effort between 4.6% and 20.4% (5^th^ to 95^th^ percentiles) of a full time technologist (%FTE) to facilitate CrAg screening workload. This reduced to between 3.2 and 11% FTE (5^th^ to 95^th^ percentiles) for Scenario II. Similarly, in Scenario III, the %FTE ranged between 3.9 and 17.1%. In Scenario IV, the expectation of much higher volumes of reflexed CrAg screening tests led to a higher %FTE range of 5.7 to 27.5%. For scenario IV, more sites would utilise automated walk-away equipment, resulting in %FTE per result that was less than the manually-based LFA testing. Fig 3 also shows the respective components of the required EIA %FTE, revealing that much of the effort required of the total FTE is comprised of the administration linked to reflexed testing and the associated review and authorisation of the results.

### CrAg cost-per-result analysis

A cost-per-result analysis was reported for defined percentiles (Table 2) across each of the four scenarios. Details of the spread sheet used to calculate the cost-per-result is outlined in S2 Table. For Scenario I, the cost-per-result varied between $5.08 and $6.41 (95^th^ to 5^th^ percentile). Similarly, for Scenarios II and III, the cost-per-result ranged from $5.63-$8.24 and $5.31-$6.90 respectively. In scenario IV the cost-per-result ranged between $4.88 (95^th^ percentile) to $5.88 (5^th^ percentile) (Table 2).

## Discussion

Recently published cost evaluation studies focused on establishing cost-per-result at a single CD4 laboratory or health facility, or cost-effectiveness of CrAg screening in a national screening initiative [7, 17]. Although these kinds of studies are useful to describe methodology and determine what components/ aspects need to be factored in when establishing the cost-per-result of a laboratory test, they do not generally address the impact of varying test volumes across a network of laboratories. The work presented in this study builds on this earlier work and extrapolates the previous cost-per-result exercise to take into account the impact of varying test volumes and use of appropriate test platforms to meet service demands. Projected volume changes, based on changing HIV/AIDS treatment guidelines [10, 18, 19] or programmatic scale-up of laboratory services needed to meet international treatment targets [11], were taken into consideration in the construction of additional scenarios presented, in an attempt to pre-empt and predict the impact of volume changes (i.e. service needs) on the cost-per-result. Our previous study reported the estimated cost-per-result in a single busy CD4 laboratory as part of a CrAg reflexed screening initiative pilot [7]. Sensitivity analysis presented in that study suggested that variation in test volumes affected cost-per-result. The current report confirms and consolidates these findings and reveals that an almost 50% increase of CrAg cost-per-result (by $2 more) or $1 cost decrease could be expected if test volumes decrease or increase by a factor of 60% respectively [7].

As expected, the LFA assay reported higher technical effort/ staffing costs due to the manual aspects inherent to the assay design, i.e. intended by the manufacturer to be used as a point of care test. In contrast, the cost of automated reflexed CrAg EIA testing is cheaper despite that EIA reagent costs are slightly higher than that of LFA. This is largely attributable to less EIA technical effort/ staffing cost which offsets the slightly higher EIA reagent costs (Table 2). Although automation substantially reduced EIA hands-on-time, the effort/ time required to manage the administration of reflex testing, including the finding of filed CD4 samples, reviewing results and re-filing samples, added effort costs. These administrative (technical) effort costs can potentially be reduced by integrating reflexed CrAg testing with CD4 testing and analysing both tests on the same testing/ analysis platform. Development of a CrAg assay for Flow cytometry [20], i.e. on the same platform currently used for CD4 testing, would facilitate full integration of CrAg screening with CD4 testing in real-time and save administrative effort. In such a system it is envisaged that, on the same flow cytometry platform, instrument software and sample preparation robotics would direct identified samples eligible for CrAg screening testing immediately after CD4 batches are completed, without removal of the sample from the platform, thus considerably reducing sample handling time.

Differences in cost-per-result of a tiered service for a national network have been reported for CD4 testing [21]. This tiered approach can be applied to the national CrAg screening program by taking advantage of a lower cost-per-result in higher throughput laboratories as part of a national service; i.e. a cross-subsidization model. The perception that providing fewer tests costs less is not necessarily true as data reported here indicate. In Scenario II, where numbers of CrAg screening tests reduced substantially in the setting of screening only newly initiated ART patients, this work reveals that an LFA could potentially cost almost $2 more per result. Although the LFA is used to support low throughput laboratories in this study, the assay was primarily designed as a point of care (POC) assay for use in a true POC/clinic context. However, testing at the POC can cost considerably more than laboratory based testing [21, 22]. To this end, the data presented here may reflect POC costs to be in the region of the 5^th^ percentile outcomes reported here. This data suggests that performing less than 10 samples per day at the POC in a health facility could result in considerably higher costs, even exceeding $8 per result, compared to the cost-per-result where testing is provided at laboratories. This is separate from the additional costs of performing a CD4 test which would also be required at the POC in the first instance [21, 22], to define whether a CrAg screening LFA should be performed at all.

Technical effort, defined as %FTE, ranged from 3.2% to 27.5% across the four test volume scenarios investigated, indicating that at any given daily threshold, CrAg testing would require no more than 1/3^rd^ of one staff member. However, this is only true if EIA is implemented for high volume testing (>30 samples per day). However, should LFA testing be introduced into all CD4 testing sites a substantial increase to the cost-per-result could be anticipated. Local cooperation with, and taking advantage of staff redundancies in sister pathology units (chemistry and microbiology) in the same testing complex, may help to alleviate the CrAg screening workload and spread the technical effort load, especially in lower volume clinical pathology sites.

A fully centralized service typically takes maximum advantage of lower cost-per-result at higher test volumes [8]. In an ideal, logistically well-organized service network with excellent sample transport systems exits, EIA platforms could be utilized across a centralized service with fewer laboratories; a projected cost saving of up to $1 per result could be unlocked. Additional cost-per-result savings could be realized and staff workload/ effort reduced (see Fig 3 for details), by implementing a rules-based auto-reviewing algorithm, ‘Auto Review’ system, where samples found to be negative on CrAg screening (95%) would be automatically authorized on the laboratory management system. This will re-focus staff effort to reviewing and authorizing only positive CrAg results, leading to a lower cost-per-result. Notwithstanding the ideal of centralized laboratory service models that maximizes on lower cost-per-result at higher volumes, prevailing largely third-world conditions limit such unified service delivery across South Africa. Local challenges to a centralized laboratory service include outdated sample transport schedules, local difficult terrain and poor road infrastructure in rural areas [8]. In some instances, opening a lower tiered laboratory service in a remote area has had better local impact, especially at the community level [23].

It is generally accepted that a public health approach, facilitating wide scale national tiered laboratory services, contain overall programmatic costs. Several aspects that play an important role in ensuring sustainability should be mentioned. The first is the importance of implementing standardized testing across tiered laboratory network. Economies of scale play an important role in containing costs. Fixed national contracts also ensure that reagent costs remain stable over time, despite fluctuating exchange rates. The importance and advantages of national procurement, tender processes and national policies to secure suppliers’ reagent prices cannot be overstated. Reagent costs can also vary when overseas manufacturers contract with local brokers to provide a local in-country service. [7]

Lastly, assessing the actual cost-per-result in the context of a national program requires an approach that addresses the realistic scenario of different laboratory service tiers with varying test volumes to meet service demands. The percentile method presented here provides an innovative but simple approach that can assist with the assessment of a cost-per-result, for a range of daily test volumes and platform choices. Such an approach facilitates assessment of the cost-per-result at varying test volumes, staff allocations and platform choices for a national program and mitigates the risk of under-pricing a test.

## Conclusion

Varying test volumes are needed to meet service demands across a national CrAg screening program, which results in a range of cost-per-result at different test volumes. The potential impact of treatment guideline changes and international treatment targets, in the context of HIV patient management and implementation of related opportunistic infection screening, should also be considered when performing costing analysis for a national program. A percentiles approach described here, enables establishing the cost-per-result for varying daily test volumes and different platforms.

### Limitations

Recent guideline recommendations propose that all HIV+ individuals should be eligible for treatment, irrespective of their CD4 count [19]. Local opinion leaders in South Africa continue to stress that baseline CD4 would still be required to assess immune deficiency, life threatening co-infections (such as Cryptococcal disease) and enable fast-tracking severely immune suppressed patients [24] onto ART, hence the basis of this report.

Lastly, the scenarios constructed here are envisaged by the authors merely to demonstrate how changes to treatment guidelines or treatment targets (like the UNAIDS 90-90-90 goals) can lead to markedly different service needs that ultimately impact upon the cost-per-result of a reflexed CrAg laboratory test. It is however possible that alternative scenarios or combinations of scenarios may need to be assessed.

### Recommendations

The following recommendations are proposed based on the results of this study:

- Adopting the percentiles approach to the cost-per-result across a national program.
- When establishing the cost-per-result of a laboratory test, the impact of volumes of tests, choice of platform and prevailing guidelines and treatment goals should be taken into account.

## Supporting information

S1 Table Workflow analysis template. This template summarizes the workflow analysis for LFA and EIA and the %FTE for each Scenario at the pre-defined percentiles.
(XLSX)

S2 Table Costing template. This template gives an example of how cost-per-result was calculate for typical volumes of LFA and EIA testing using scenario I or status quo, with reference to volumes, quoted test costs, staff costs and exchange rate used.
(XLSX)
